# Examining the implementation of the Icelandic model for primary prevention of substance use in a rural Canadian community: a study protocol

**DOI:** 10.1186/s12889-020-09288-y

**Published:** 2020-08-14

**Authors:** Tanya Halsall, Lisa Lachance, Alfgeir L. Kristjansson

**Affiliations:** 1 Youth Research Unit, The Royal’s Institute of Mental Health Research affiliated with the University of Ottawa, 1145 Carling Ave, Ottawa, Ontario K1Z 7K4 Canada; 2 Faculty of Health, Dalhousie University, 6299 South St, Halifax, NS B3H 4R2 Canada; 3 School of Public Health, RC Byrd Health Sciences Center, West Virginia University, 64 Medical Center Drive, Morgantown, WV 26506 USA; 4 Icelandic Center for Social Research and Analysis, Reykjavik University, 1 Menntavegur, 101, Reykjavík, Iceland

**Keywords:** Substance use prevention, Mental health promotion, Bioecological model, Positive youth development, Implementation research, Collaboration, Community-based, Youth engagement, Evaluation, Case study

## Abstract

**Background:**

The Icelandic Prevention Model (IPM) is a collaborative upstream model that was designed to influence risk and protective factors related to substance use within the community, school, peer and family contexts. By engaging whole communities, the IPM has been found to be effective in reducing youth substance use behaviours across Iceland. As an extension to the IPM’s participatory approach, this research will examine how youth involvement can enhance outcomes. In addition, this research will evaluate whether the IPM approach is beneficial for mental health promotion and general youth wellbeing.

**Methods:**

The present research protocol applies the bioecological model within a participatory mixed-method case study design to examine the implementation of the IPM in a rural community in Canada. This study was designed to identify whether the Icelandic substance use prevention model is effective in reducing substance use and promoting mental health and development for Canadian youth. It will also explore how to engage youth within the approach and how this adaptation influences implementation and outcomes.

**Discussion:**

The findings from this study will contribute to our understanding of upstream prevention of youth substance use and will be used to support scaling of the IPM across Canada.

## Background

Over 40% of Canadian secondary students report consuming alcoholic beverages in the past 12 months [1]. When consumption of alcohol is excessive, it is associated with significant risks of negative outcomes for youth including self-harm, vehicle accidents, substance use disorders, school performance issues and school dropout [2]. Likewise, the proportion of opioid-related deaths among youth aged 15–24 years in Ontario has increased 10th fold over the last 15 years, or from 1.1–11.6% [3]. In the context of Canada’s opioid crisis, together with the legalization of cannabis for recreational use, Canada’s Chief Public Health Officer [2] has called for a greater focus on the prevention of problematic substance use among youth and highlighted the need to use youth- and community-driven interventions to address risk and protective factors. The Icelandic Prevention Model (IPM) is a collaborative model that uses a community-based participatory approach to identify risks and mobilize protective factors to prevent future substance use in youth [4, 5]. Rather than targeting individual behaviours, the IPM considers broader contextual influences on the lives of youth including family, peers, school and community. In Iceland, trend analyses identified a national decline in youth substance use including a 46% reduction in the proportion of youth getting drunk during last 30 days prior to each annual survey [4]. In another trend analysis that examined a 17 year period, 30-day alcohol intoxication declined from 29.6 to 3.6% [6]. In addition, researchers applied a quasi-experimental study to compare communities that received the IPM with control communities [7]. They found that parental monitoring and youth sport participation increased in intervention communities while time spent in unstructured activities and unsupervised social gatherings decreased when compared with controls. Use of alcohol and 30-day intoxication rates also decreased in comparison with control communities.

The IPM is now being implemented worldwide as a collaboration between the Icelandic Centre for Social Research and Analysis (ICSRA) and local entities through the Planet Youth platform (see www.planetyouth.org). However, it has not yet been applied in Canada, which given the geographic distribution of communities and variations of community identity, means that adaptations may be necessary to achieve maximal effectiveness. Moreover, although the model is community driven, previous iterations have not involved youth in the development and implementation of specific interventions. Finally, potential impacts on mental health and wellbeing beyond substance use have not yet been examined formally although the idea of scaling the model to include mental health have been raised before [8]. This protocol describes a study that applies the ecological model within a case study approach to examine IPM adaptation, implementation and related outcomes in order to inform scaling across Canada.

### Positive youth development and substance use prevention

Adolescence is a critical stage in the life course and events during this period have the potential to significantly influence developmental trajectories and future individual success [9]. During adolescence, there is an increase in outside sources of influence on healthy development, including peers and the media [9]. As a result of brain maturation, there is also a concurrent increase in impulsivity and risk-taking [10] while executive functioning and self-regulation skills are not yet fully developed [11]. Placing positive youth development at the core of an intervention involves recognition of the need to examine the connection between young people and their environment holistically [12–14]. This includes acknowledging that risky behaviours often co-occur [15], and additively increase the likelihood of diminished developmental outcomes, just as both personal and social assets function to enhance positive developmental outcomes [16, 17]. Taking a holistic and integrated perspective, it is hypothesized that the IPM intervention, which is designed to influence a range of risk and protective factors, may have a broader impact on youth wellbeing beyond the prevention of substance use behaviours.

### Bioecological model

The bioecological model provides a useful lens to examine multi-level system interventions to examine individual development. The model combines four major components: 1) process, 2) person, 3) context, and 4) time [18]. *Process* is the most fundamental concept within the model and represents the increasingly complex reciprocal influence between a developing individual and their environment. The *person* component represents both individual agency and outcomes. The concept of *time* signifies the dynamic temporal nature of development as the well as the historical atmosphere. Finally, *context* represents the embedded systems that influence development and includes micro-, meso-, exo- and macro-systems. Within a youth-driven participatory approach, the ecological concepts of *person* and *process* can be applied as a guiding framework to understand and integrate youth perceptions of the intervention and youth influence on project development.

### Youth engagement

Youth engagement entails the participation of youth in meaningful activities that integrate their perspectives into “the institutions and decisions that affect their lives” [19] p. 341). This approach was initially stimulated by an increased recognition of the rights of children emanating from the UN Convention on the Rights of the Child [20]. Since then, it has been acknowledged that engaging youth is an essential component of effective youth development programs and policy, and increases the likelihood of positive outcomes for youth, organizations and systems [21].

The majority of substance use prevention efforts have not included youth voice which has often resulted in interventions that are not congruent with youth lived reality [22]. Including the youth lens strengthens the capacity to examine how youth experience an intervention and enables identification of the mechanisms of influence from the intervention [23]. Youth contributions may also enhance the interpretation of survey findings by increasing the relevance of strategic directions, improving feasibility of methods, and providing a more nuanced interpretation of findings. Youth engagement has also been applied to evaluation research [24–26], and youth participatory evaluation enhances quality, reliability and validity of data [27]. Historically, the IPM has primarily been operated as an ecological model based in classical theories of adolescent deviance [28]. As such, the IPM assumes that the environment is the primary producer of deviant behaviors. However, recognizing the potential value of including youth perspectives within the IPM process, Planet Youth Lanark County has incorporated a youth engagement process, and youth will be a major stakeholder in adapting and implementing interventions stemming from the model. These youth stakeholders will also be involved as co-researchers within the participatory evaluation.

#### Context

Planet Youth Lanark County (PYLC) originated when a community interest group was created in response to concerns about opioid risks in the community. They identified the IPM as a possible approach to support the objective of protecting their children and youth. This led to the formation of the PYLC Steering Committee and a formal partnership with the ICSRA to support the first implementation of the IPM in Canada. Currently, the PYLC Steering Committee has engaged key stakeholders to support the implementation of the IPM model, including United Way East Ontario, Open Doors for Lanark Children & Youth, the Leeds, Grenville & Lanark District Health Unit, the Royal’s Institute of Mental Health Research, the Catholic District School Board of Eastern Ontario, and the Upper Canada District School Board.

The IPM approach incorporates five principles that guide design of community-specific strategies: 1) apply a primary prevention approach, 2) engage community action and public school involvement, 3) engage stakeholders using high-quality data, 4) integrate researchers, policy makers, practitioners, and community members, and 5) align the scope of the solution with the nature of the problem [28]. At the outset, a needs assessment is conducted through youth surveys, and the findings are disseminated to inform a tailored prevention strategy that leverages existing community structures to promote sustainability [29]. As a component of the intervention, surveys will be distributed to all Grade 10 students in the participating Lanark school boards [29]. Survey measures capture risk and protective factors in relation to individual, family, peer, school, leisure time activities and community characteristics as well as baseline information regarding substance use behaviours and mental health and wellbeing. The data provide diagnostic information to assist the community to align potential solutions. Based on the findings, community-driven goal setting and strategic planning is conducted and Community Coalition teams are formed to implement the plans.

##### Planet Youth Lanark County Implementation.

The PYLC has incorporated an innovative deviation from the traditional IPM model, in that they will include community youth as a key stakeholder from the outset. Within youth-adult partnerships, it is important to ensure that youth engagement principles are embedded at the system-level [30]. This includes the development of policies and procedures as well as capacity building for partners that supports the inclusion of youth perspectives. In support of the youth engagement strategy, community public health nurses will facilitate a targeted recruitment of students to form a Youth Advisory Group. These youth will be involved in all aspects of the intervention design and implementation.

As a significant component of the IPM intervention, a baseline survey will be administered in October 2020 that examines a range of risk and protective factors, substance use behaviours and mental health indicators. The IPM model applies a passive consent process in the surveys, therefore participation rates typically exceed 85% of the total student population within selected grades. In Lanark County the total grade 10 population within both school boards is approximately 900 students, therefore, approximately 765 students will likely participate. Analysis and dissemination of the findings will occur in December 2020. Findings will be provided to each school and municipality, and in particular, the correlations among risk/protective factors and substance use behaviours within this population. These data will inform the design of the intervention within each municipality. In addition to the metrics typically used at the outset of the IPM process, the PYLC team has completed a review of positive mental health and well-being indicator frameworks and expanded the survey for implementation in Lanark County. The intent is to examine whether the intervention has an effect over time on mental health and wellbeing, in addition to substance use behaviours. The partnership with the ICSRA will extend over the next 5 years and survey data collection will be repeated at years three and five to examine changes in risk and protective factors, substance use behaviours and mental health indicators.

#### Research objective

The proposed research project applies the bioecological model to examine a substance use prevention model for youth that integrates considerations related to the individual, their context, and the interaction between them. This research applies a mixed method case study approach to examine IPM adaptation, implementation and related outcomes. Recognizing that the IPM intervention already includes a participatory process that engages multiple stakeholders in decision-making, the research will entail multiple components, including a needs assessment, youth participatory evaluation, implementation evaluation, and outcome evaluation. Specific evaluation questions include: 1) How can youth voice be integrated into the IPM model to inform development, implementation and evaluation of interventions? 2) How do community youth experience the IPM interventions? 3) Has the IPM been implemented with fidelity? 4) What adaptations are needed to implement the IPM in a rural Canadian community? 5) Does the IPM have an impact on youth substance use, mental health and wellbeing outcomes?

The research design integrates qualitative methods, including participant observation, semi-structured interviews and implementation fidelity assessment as well as quantitative methods (student survey, social network analysis). This protocol was written using the Standard Protocol Items: Recommendations for Interventional Trials guidelines.

## Method

**Fig. 1:**
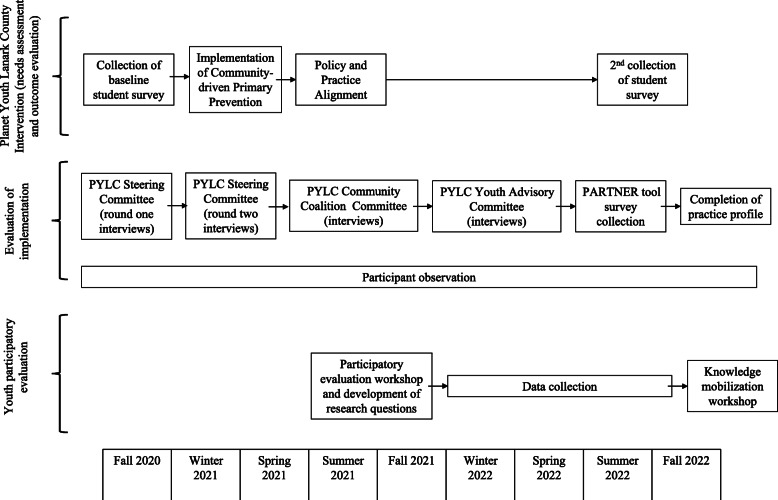
Planet Youth Lanark County Case Study Overview. The figure displays each component of the study and presents the timeline for implementation

Pragmatists apply the methods that work to solve a specific problem [31] and allow contextual requirements to drive method choices [32]. Since this is a study of a complex set of several interventions that are designed to examine context and dynamic process with the intent to support implementation, a mixed-method case study design will be employed guided by a pragmatic research paradigm (see Fig. 1 for study overview). Case study involves the in-depth examination of a delineated unit [33, 34]. Case study methods are useful when studying complex interventions and can be used to describe interventions and context as well as to explain their functions [35]. Researchers have argued that studies designed to examine complex, system-level initiatives “must be augmented by the study of how we can best deal with uncertainty, unpredictability and generative causality. For this, we need research designs and methods that foreground dynamic interactions and emergence – most notably, in-depth, mixed-method case studies that can act as concrete, context-dependent exemplars” [36](p.2). Case study has been successfully applied to examine implementation within a variety of health-related settings, including the implementation of evidence-based guidelines [37], researcher-clinician collaborative projects [38], decision-support interventions for patients [39], quality improvement processes [40] and children’s mental health and social service organizations [41]. In this study, PYLC is the identified case with the purpose of examining in-depth implementation processes and outcomes. Such an understanding can form the basis of scaling the program across Canada.

### Needs assessment and outcome evaluation

A partnership agreement has been signed with the ICSRA in order to support the implementation of the IPM in Lanark County. This includes the collection of baseline population-level surveys to examine risk and protective factors that influence problematic substance use. Surveys will be distributed to all Grade 10 students from both local school boards using a passive consent process [29, 42]. The data collection will be used as the basis for a needs assessment. This is achieved by capturing existing levels of risk and protective factors to provide data-driven diagnostics and align potential solutions with these findings. The data also serve as a baseline as the survey measures levels of substance use and wellbeing. The findings will be distributed to schools and communities within 3 months and will be repeated at years three and five to support on-going adaptations and outcome evaluation. In addition to standard items, new survey questions have been included that expand on individual and contextual strengths. These were developed through a review of current frameworks examining positive mental health and wellbeing, including the Child/Youth Index of Wellbeing [43], the Health Behaviour in School-aged Children survey [44], the Public Health Agency of Canada’s Positive Mental Health Surveillance Indicator Framework [45], the Developmental Assets Framework [16], the Canadian Index of Wellbeing [46], the Catholic District School Board of Eastern Ontario Building Resiliency and Positive Mental Health initiative [47] and the Child and Youth Resilience Measure [48].

As the data will be population-based, survey reports will provide descriptive statistical analyses and correlations to describe relationships between risk and protective factors and substance use and mental wellness indicators. Findings from the survey data will also be analyzed by gender and other demographic variables in order to distinguish outcome patterns [49]. Findings will highlight population changes in substance use behaviours, levels of mental health and related risk and protective factors such as engagement in organized activities, parental connections with peers and shifts in school climate. In addition, demographic variables will serve to highlight which populations experience differential outcomes associated with factors such as gender, school catchment and peer group characteristics.

### Social network analysis

The PARTNER tool [50, 51] was designed to conduct social network analysis in order to examine how community partners collaborate. The PARTNER tool includes a validated survey and analysis software designed to examine partner interconnections. The survey includes questions that examine respondent motivation for participating in the network, resource contributions, length of time of involvement, nature of contributions to the network, perceived level of success and major outcomes. The survey also contains relational questions that capture information about specific relationships among partners with respect to frequency of interaction, perceptions of trust and value, and level of collaboration. The PARTNER tool analyzes this information to calculate network density and degree of centralization, which relates to the extent of overall partnership development and pattern of relationships. The PARTNER tool will be used to conduct a social network analysis to examine how community partners collaborate to implement the PYLC intervention. Representatives from each committee, municipality, school, and participating community organizations will be invited to complete the survey. Within this initiative, there are currently three committees, nine municipalities, seven schools and five partner organizations for a total of 24 survey respondents. Participants will receive emails inviting them to complete the survey in May of 2021. Several reminders will be sent to ensure a high response rate.

Descriptive analyses from the PARTNER tool data will identify interactions between partners and the nature of partnership among committees and organizations across the network (i.e. interactions are cooperative, coordinated or integrated). A network map will be created and analysis of reciprocal perceptions of relationships and committee/organizational characteristics among collaborating network members will be completed. Whole network scores will be calculated with respect to density (amount of connections), centralization (configuration of the connections) and overall trust by aggregating responses from across all partners. This data will provide an intricate view of how partnerships are created and how they might influence the effectiveness of the overall initiative.

### Participant observation

The lead author (Halsall) has been supporting the PYLC Steering Committee meetings since the spring of 2019 and will continue through the duration of this research (fall 2022). In addition, Halsall also participated in key community events, including the Municipal Drug Strategy Steering Committee Strategic Planning Day, the IPM training events and relevant working group meetings. Going forward, Halsall will participate in strategic planning meetings and community events and will also play a lead role in the dissemination of findings to the community. Field notes and meeting documentation will be used to capture on-going implementation processes.

### Implementation fidelity assessment

The overall objective of implementation science is to support the uptake of evidence into practice [52]. Initially, implementation was conceptualized as linear and mechanistic, however this approach does not align well within complex systems [52]. The National Implementation Research Network has developed a variety of tools that can be applied to examine complex collaborative efforts. The practice profile [53] is designed to describe the critical components of a complex intervention as well as the expected, developmental and unacceptable practice variations. This allows systematic tracking of the extent of alignment with original intentions, and the modifications that occurred. Critical components of this aspect of the research will be based on the five guiding principles and ten core steps to implementation and will be elaborated using published implementation science findings and in consultation with the ICSRA. The practice profile will be completed in November of 2021 to assess fidelity of the PYLC implementation to the IPM model. The profile will be completed using data from participant observation, social network analysis data and the interviews. For example, one of the guiding principles specifies that the approach integrate researchers, policy makers, practitioners, and community members into a unified team. This will be verified by examining stakeholder participation, including committee meeting attendance, network map connections and interview data.

A scoring system was created to examine implementation data from the practice profile and ratings assigned with respect to fidelity to the model. Scores from each guiding principle and core steps will be ranked as a 3 – implementation as expected, 2 – developmental implementation, 1 – unacceptable implementation. Sub-scores will be averaged to obtain an overall score ranging from high fidelity [3] to low-fidelity [1]. Scores will also be integrated with dissemination of year 3 survey data to inform adaptations going forward.

### Semi-structured interviews

Semi-structured interviews will be conducted with PYLC Steering Committee members in the summer of 2020 to explore the background and initial development of PYLC. The PYLC Steering Committee is the over-arching governance structure for the PYLC initiative and they are responsible for bringing the IPM to Lanark County and for the oversight of the overall implementation. These interviews will examine the processes that were evoked during the initial steps of considering the IPM model, including adherence to the five guiding principles. Once the intervention strategy is identified and rolled out, a second round of interviews will be conducted with the Steering Committee members to explore the process of developing the intervention and to review fidelity with respect to the later steps delineated in the IPM implementation process. In addition, interviews will be conducted with Community Coalition members (the implementation teams) and Youth Advisory Committee members.

Interview guides will tap into key stakeholder perspectives with respect to context, implementation and outcomes. Interview questions will focus on their roles, lessons learned, rationale for the community strategy, processes for dissemination of findings, key factors that influenced decisions, successes and challenges. A sub-set of interviews in the Steering Committee and Community Coalition questions were adapted from critical questions identified in the Quality Implementation Framework [54] and explore implementation components within the IPM [29] (see Additional files 1, 2, 3). A subset of questions in the Youth Advisory interviews examine youth perceptions of substance use issues, experiences of risk and protective factors identified within the surveys and insights related to potential mechanisms of influence (see Additional file 4). Currently, there are about 15 active members of the Steering Committee. Community Coalition and Youth Advisory member counts will likely be 10–12 for each committee. Semi-structured interviews are estimated to be one-hour in length. The goal is to interview all committee members, and they will be recruited during committee meetings.

**Fig. 2:**
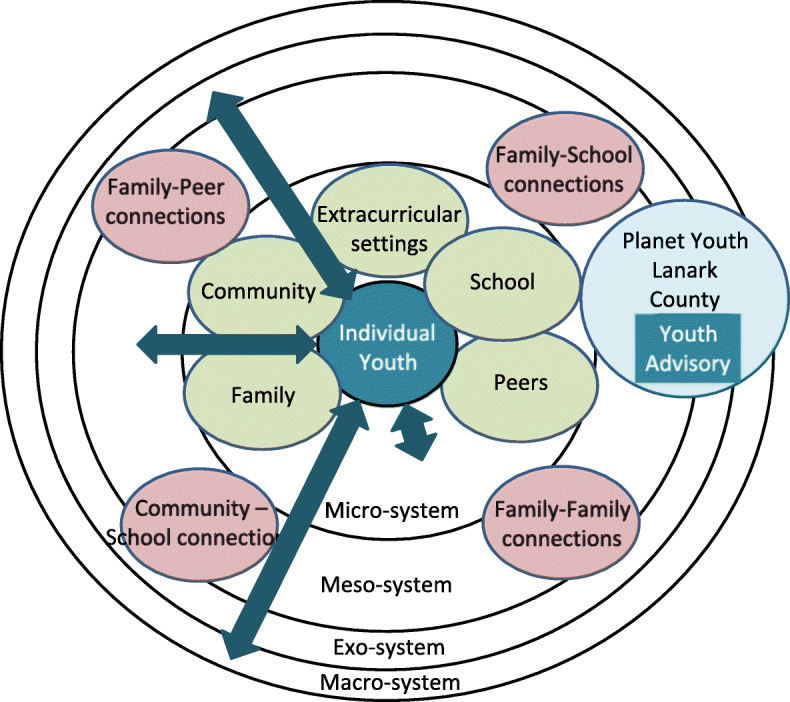
Bioecological model applied to the Planet Youth Lanark County intervention. The figure displays the multiple components and target areas of the Planet Youth Lanark County intervention embedded in the system that is implicated within the bioecological model

The qualitative interviews will be transcribed and sent to the participants for review in order to receive a member check and to identify if participants would like to make any changes or adaptions. Thematic analysis will be conducted on the transcripts [55, 56]. This involves the following steps: 1) familiarization with the data, 2) generation of initial codes, 3) identification of themes, 4) revision of themes, 5) definition and specification of themes and 6) development of the report. Data will be analyzed using the QSR NVivo software. A second coder will provide inter-rater reliability by coding a sub-set of interviews. Both coders will discuss and refine codes and higher order themes until consensus is reached. Meyer and Ward [57] suggest that an isolated deductive approach has limitations as it cannot capture new insights, while a standalone inductive approach does not take into account the body of knowledge that preceded it. This design applies a deductive and inductive approach, whereby some interview questions are derived from implementation science and the intervention theory of change and others are more exploratory examining context and possible mechanisms of influence. Consequently, codes that relate to the bioecological model and intervention concepts will be used to examine the data deductively, while emergent issues will be identified inductively [58]. Preliminary analyses will be presented to the Steering Committee and Youth Advisory in order to elicit their perceptions and interpretations of the findings. Figure 2 illustrates how Planet Youth Lanark County intervention components overlap with bioecological concepts and processes.

### Participatory youth evaluation

Youth engagement principles should be applied to the development of policies and procedures as well as capacity building for partners that supports the inclusion of youth perspectives [30, 59]. Therefore, this project involves multiple levels of youth participation that are integrated within each stage of the initiative. This strategy will involve two youth leads who will sit on the PYLC Steering Committee. They will support engagement and communication with community youth and will co-facilitate youth-focused workshops. To form the youth advisory, individuals will be identified who represent diversity based on the following factors: 1) community location, 2) ethnicity, 3) age, 4) gender, 5) LGBTQ identity, 6) socio-economic status and 7) Indigenous identity. A diversity survey will be collected with advisory youth that will include items that capture participant demographics with respect to the key perspectives. Additional recruitment strategies will be implemented to ensure all relevant perspectives are included.

A participatory evaluation workshop will be implemented to identify research questions and methods of interest and will present principles of evaluation and participatory research and a discussion to reflect on major issues of interest related to the intervention. Congruent with participatory approaches, the research team has successfully collaborated with youth in the past to co-create research questions and methods [26, 30, 60, 61]. Research questions have often focused on current issues affecting youth. For example, experiences related with a rural lifestyle, including access to amenities, vocational opportunities and transportation may become key issues of focus. Methods can often be adapted to integrate youth-friendly activities such as games, visual arts and technology. A knowledge mobilization workshop will focus on stakeholder analysis [62] and youth will explore possible strategies for applying findings locally and beyond Lanark County to support desired outcomes.

### Note on recent developments related to the COVID-19 pandemic

This project has been delayed as a result of recent public health measures taken to restrict the spread of the coronavirus. The timeline has been shifted by six months and some data collection procedures may need to be adjusted to occur online in order to accommodate for social distancing (e.g. Steering Committee interviews). In addition, the ICSRA is conducting global consultations in order to make adaptations to the student survey to support community response to COVID-19-related impacts. As such, the student survey will also be used to inform the development of community supports when it is distributed in the fall.

## Discussion

This research program will, for the first time, examine the implementation of the IPM: 1) within the Canadian context, 2) combined with a youth-driven approach 3) with a focus on implementation to better understand how the intervention works, and 4) with a strategy to measure mental health and wellbeing outcomes. There are a range of aspects that will be applied within this study that will contribute to methodological rigour (see [63]). The approach is informed by theory and combines several methods and tools to enhance design. Community partner and youth perspectives are holistically integrated into the design and will strengthen the feasibility of methods and relevance of findings. A deductive-inductive analytic process will be used that involves multiple coders. In addition, the study demonstrates the quality criteria of credibility, significant contribution and topic worthiness (see [64]).

The proposed initiative will also make several significant practical contributions, including 1) a better understanding of the underlying mechanisms of influence within the IPM, 2) potential enhancement of the health and well-being of Lanark County youth, and 3) the development of an evaluation framework and collection of implementation findings to support national scaling of the IPM intervention. This research will contribute new scientific knowledge with respect to theory-driven evaluation, mental health promotion at the population level, and youth engagement in design and implementation of complex initiatives.

## Supplementary information

**Additional file 1.** Steering Committee interview guide – Spring 2020. Semi-structured interview guide questions to be used with the Steering Committee after the completion of first four core steps of the Icelandic Prevention Model

**Additional file 2.** Steering Committee interview guide – Spring 2021. Semi-structured interview guide questions to be used with the Steering Committee after the completion of second four core steps of the Icelandic Prevention Model.

**Additional file 3.** Community Coalition interview guide. Semi-structured interview guide questions to be used with the Community Coalition Committee.

**Additional file 4.** Youth Advisory interview guide. Semi-structured interview guide questions to be used with the Youth Advisory.

## Data Availability

Data sharing is not applicable to this article as no datasets were generated or analysed during the development of this protocol. Full copies of interview guides are provided.

## Abbreviations

IPM: The Icelandic Prevention Model
ICSRA: Icelandic Centre for Social Research and Analysis
PYLC: Planet Youth Lanark County
