# Supplementary material for: Examining the implementation of the Icelandic model for primary prevention of substance use in a rural Canadian community: a study protocol
Source: BMC Public Health. 2020 Aug 14;20:1235. doi: 10.1186/s12889-020-09288-y (PMC7426669; doi:10.1186/s12889-020-09288-y)
Supplement: Supplementary file 1 — Additional file 1. Steering Committee interview guide – Spring 2020. Semi-structured interview guide questions to be used with the Steering Committee after the completion of first four core steps of the Icelandic Prevention Model [file 12889_2020_9288_MOESM1_ESM.docx]

Steering Committee interview guide – Spring 2020

**Introduction**

Can you describe your role on the Planet Youth Lanark County Steering Committee? Why have you become involved?

- Length of involvement?
- Perspective represented?

In terms of the IMP model, we have now completed the first three steps:

1. Local Coalition Identification, Development, and Capacity Building
2. Local Funding Identification, Development, and Capacity Building
3. Pre–Data Collection Planning and Community Engagement

Are there any key lessons learned that you would like to share about this process? Please describe.

- - *Probes: Emergent components and adaptations? Critical events? Successes? Challenges? Most important factor? Anything we can’t function without?*

What impacts have you perceived that have resulted from the work of PYLC so far?

**Critical steps (1,2,3&5) in implementation, important questions to answer at each step in the Quality Implementation Framework**

**Conducting a needs and resources assessment:**

- Why was PYLC formed?
- What problems or conditions will PYLC address (i.e., the need for the innovation)?
- Who will benefit from improvement efforts?

**Conducting a fit assessment:**

- Does the IMP fit for Lanark County?
- How well does the IMP match the:
  - Identified needs of the community?
  - Cultural preferences of groups who participate in services provided by the community?

**Conducting a capacity/readiness assessment:**

- To what degree does the community have the will and the means (i.e., adequate resources, skills and motivation) to implement the IMP?
- Is the community ready for this change?

**Obtaining explicit buy-in from critical stakeholders and fostering a supportive community climate:**

- What level of buy-in has the PYLC received from:
  - Leadership with decision-making power in the community?
  - From individuals who will likely deliver the IMP?
  - The local community?
- How has PYLC dealt with important concerns, questions, or resistance to the IMP?
  - What barriers has the Steering Committee experienced in establishing the PYLC?
- What local champion(s) have ben identified to support the PYLC effort?
  - Are there one or more individuals who can inspire and lead others to implement the IMP and its associated practices?
  - Are there any perspectives that are missing from the PYLC Steering Committee that should be recruited? Please describe.

Is there anything else you would like to share that we haven’t had a chance to discuss?

De-brief
