# Supplementary material for: Examining the implementation of the Icelandic model for primary prevention of substance use in a rural Canadian community: a study protocol
Source: BMC Public Health. 2020 Aug 14;20:1235. doi: 10.1186/s12889-020-09288-y (PMC7426669; doi:10.1186/s12889-020-09288-y)
Supplement: Supplementary file 2 — Additional file 2. Steering Committee interview guide – Spring 2021. Semi-structured interview guide questions to be used with the Steering Committee after the completion of second four core steps of the Icelandic Prevention Model. [file 12889_2020_9288_MOESM2_ESM.docx]

*Steering Committee interview guide – Spring 2021*

At this stage, PYLC has completed Steps 4-9 in the critical steps of the IMP:

- Data Collection and Processing
- Enhancing Community Participation and Engagement
- Dissemination of Findings
- Community Goal-Setting
- Policy and Practice Alignment
- Child and Adolescent Immersion in Primary Prevention

How have you been involved in the process?

From your perspective, what were some key lessons learned from this experience?

What were the major successes in the implementation process?

What were the major challenges in the implementation process?

How were survey findings disseminated to the community?

How were prioritized intervention strategies selected?

Why were the prioritized intervention strategies selected?

What impacts have you perceived that have resulted from the work of PYLC so far?

**Critical steps (6-9,11,13) in implementation, important questions to answer at each step in the Quality Implementation Framework**

6. Building capacity:

What capacity has been developed to ensure the intervention will be implemented with quality?

- Partnerships developed/leveraged
- Communication strategy
- Funding established
- Policy and practice alignment

7&9. Intervention delivery and creating Community Coalition teams:

How were the Community Coalition teams developed?

What are the specific roles and responsibilities of Community Coalition members?

Who is supporting the delivery of the selected community intervention strategies?

- How have their roles been back-filled/supported?

What is the community liaison role in supporting implementation?

Who else is supporting the individuals who are implementing the intervention?

8. Effective pre-innovation training

How were the guiding principles and critical steps of the IMP communicated?

11. Technical assistance/coaching/supervision:

What technical assistance has been provided to help the community deal with practical problems related with the intervention? Please describe.

13. Supportive feedback mechanism

What is the process through which key findings from implementation are communicated, discussed, and acted upon? Please describe.

Is there anything else you would like to share that we haven’t had a chance to discuss?

De-brief
