# Supplementary material for: Examining the implementation of the Icelandic model for primary prevention of substance use in a rural Canadian community: a study protocol
Source: BMC Public Health. 2020 Aug 14;20:1235. doi: 10.1186/s12889-020-09288-y (PMC7426669; doi:10.1186/s12889-020-09288-y)
Supplement: Supplementary file 4 — Additional file 4. Youth Advisory interview guide. Semi-structured interview guide questions to be used with the Youth Advisory. [file 12889_2020_9288_MOESM4_ESM.docx]

*Youth Advisory interview guide*

*Intro*

- *How did you get involved in the PYLC Youth Advisory?*
- Why have you become involved?
  - Probes:
    - Time length of involvement
    - Perspective represented

*Perceptions of community issues*

- *What are the typical characteristics of Lanark County youth who experience challenges with substance use?*
- *Based on your experience, what do you think influences this behaviour?*

*Perceptions about survey data 🡪 Re-visit main findings for interviewee school/community*

- *Why do you think the identified risk factors were identified as an issue at your school/in your community?*
- *How do these risk factors affect you?*

*Intervention*

- *How, if at all, has PYLC changed things for you? Your peers? Other youth?*
- *How were youth involved in PYLC? Do you feel that youth perspectives influenced the overall PYLC approach? How?*
- *What challenges, if any, did you experience in supporting PYLC?*
- *What successes, if any, did you experience in supporting PYLC?*
- *How, if at all, might the community-identified strategy potentially influence youth substance use behaviours?*
- *Is there anything else you want to discuss about your experience on the PYLC youth advisory?*

De-brief (not for research)

- How did you find the interview?
- Do you have any questions for me?
